# Autophagy induction and PDGFR-β knockdown by siRNA-encapsulated nanoparticles reduce chlamydia trachomatis infection

**DOI:** 10.1038/s41598-018-36601-y

**Published:** 2019-02-04

**Authors:** Sidi Yang, Yannick Traore, Celine Jimenez, Emmanuel A. Ho

**Affiliations:** 1 University of Manitoba, College of Pharmacy, 750 McDermot Ave, Winnipeg, Manitoba R3E 0T5 Canada; 2 Laboratory for Drug Delivery and Biomaterials, School of Pharmacy, University of Waterloo, 10 Victoria St S, Kitchener, Ontario N2G1C5 Canada

## Abstract

C. trachomatis is the most common sexually transmitted bacterial infection in the world. Although the infection can be easily controlled by the use of antibiotics, several reports of clinical isolates that are resistant to antibiotics have prompted us to search for alternative strategies to manage this disease. In this paper, we developed a nanoparticle formulation (PDGFR-β siRNA-PEI-PLGA-PEG NP) that can simultaneously induce autophagy in human cells and knock down PDGFR-β gene expression, an important surface binding protein for C. trachomatis, as a strategy to reduce vaginal infection of C. trachomatis. PDGFR-β siRNA-PEI-PLGA-PEG NP significantly induced autophagy in human vaginal epithelial cells (VK2/E6E7) 48 hr post treatment by improving autophagic degradation activity without causing inflammation, apoptosis or any decrease in cell viability. Beclin-1, VPS34 (markers for initiation stage of autophagy), UVRAG, TECPR-1 (markers for degradation stage of autophagy) were found to be significantly upregulated after treatment with PDGFR-β siRNA-PEI-PLGA-PEG NP. Furthermore, PDGFR-β siRNA-PEI-PLGA-PEG NP decreased PDGFR-β mRNA expression by 50% and protein expression by 43% in VK2/E6E7 cells 48 hr post treatment. Treatment of cells with PDGFR-β siRNA-PEI-PLGA-PEG NP significantly decreased the intracellular C. trachomatis and extracellular release of C. trachomatis by approximately 65% and 67%, respectively, *in vitro* through augmenting autophagic degradation pathways and reducing bacterial binding simultaneously.

## Introduction

Chlamydia trachomatis (C. trachomatis) is a gram-negative bacterium that preferentially infects epithelial cells of the genital tract and causes the most common sexually transmitted bacterial infection in the world^1^. Unfortunately, about 80% of chlamydial infections in women are asymptomatic or with minimal symptoms, but if left untreated, the infection can lead to pelvic inflammatory disease, tubal infertility, ectopic pregnancy, premature delivery, and increased risk of developing cervical carcinoma. Furthermore, chlamydia infection can be passed to exposed newborns during birth resulting in conjunctivitis and possibly interstitial pneumonia^2^. The infection can also affect men, but it usually appears symptomatic and manifests as urethritis, and if left untreated, the infection can lead to epididymitis and proctitis^1^.

C. trachomatis is an obligate intracellular bacterium with two distinct forms, the infectious elementary body (EB) and the replicative reticulate body (RB) during its life cycle. Pathogenesis of chlamydia infection in the female genital tract begins with initial binding of EB to genital epithelial cells, and is followed by contiguous endocytosis through a membrane-bound compartment, inclusion^3^. After internalization, inclusion helps EB to rapidly escape the host endo-lysosomal pathway to avoid being degraded by the host defense system. At the same time, EB accomplishes the transformation into RB and begins to initiate bacterial protein synthesis. Newly synthesized inclusion membrane proteins assist the replication of RB by collecting and supplying nutrients from the host’s golgi^3^. As RB propagates and accumulates, the life cycle enters the late phase, in which late-phase effectors and EB effectors are being synthesized and the differentiation of new EB from RB is accomplished shortly afterwards. Eventually, newly produced EB leaves the host cells via extrusion (a process where a cell exports large particles or organelles through its cell membrane to the outside) or lysis to establish future infections^3^.

C. trachomatis is found to be able to infect various cell types *in vitro* and uses several receptors for binding to the host cells^4^. Initial binding of chlamydia starts with a primary reversible electrostatic interaction between EB and the host cell’s heparan sulfate receptor, followed by an irreversible secondary binding to other possible receptors such as the platelet derived growth factor receptor-β (PDGFR-β)^5^. Elwell *et al*. have shown that PDGFR-β knockdown with RNA interference decreased cell-associated bacteria by 50%. PDGFR plays an important role in vascular development and a mouse model has indicated that deletion of PDGFR-α or PDGFR-β causes no vascular defects but deletion of both disrupted the vascular development in yolk sac^6^. Further studies reported that an FDA-approved PDGFR inhibitor, imatinib, for the treatment of Philadelphia chromosome-positive chronic myelogenous leukemia and gastrointestinal stromal tumors demonstrated minor adverse effects^7^. Therefore, knocking down PDGFR-β alone as a local therapy is expected to have negligible side effects. As a result, PDGFR-β can be potentially utilized as a therapeutic target to prevent and minimize chlamydia infection of host cells^5^.

Autophagy is a self-degradative process for providing energy under stressed conditions, degrading and recycling long-lived proteins and damaged organelles as well as playing an important role in eliminating intracellular pathogens (i.e. viruses and bacteria)^8^. Autophagy has been divided into three types: macroautophagy, microautophagy and chaperone-mediated autophagy. Among the three types, macroautophagy appears to play an important role in protecting cells against microbial infection^9^. Macroautophagy is composed of two subsequent stages, the initiation stage and the degradation stage^9^. During the initiation stage, a membrane called phagophore is invaginated from cell membranes and elongated to sequester components targeted for degradation, forming an enclosed double-membrane vesicle called autophagosome. This stage is subsequently followed by the degradation stage, during which the autophagosomes will then fuse with the acidic, enzyme-enriched lysosome, forming the degradative vesicle, autolysosome. During the fusion, only the outer layer of the double membrane of autophagosome fuses with lysosome while the inner layer becomes degraded, resulting in a single-membrane autolysosome. After that, the components inside of the autolysosomes are degraded into amino acids, fatty acids, nucleic acids and so forth for recycle and reuse^8^. Detecting and identifying the bacterial components that are attached to or inside of the cytoplasm of mammalian cells is a key process for initiating macroautophagy. Recognition of bacterial lipopolysaccharide by toll-like receptor 4^10^, detection of bacterial peptidoglycan by NOD-like receptors^11^ as well as the identification of intracellular bacteria by sequestosome-1-like receptors^12^ are identified pathways for the initiation of autophagy in the defense against bacterial infection. Various regulatory proteins (e.g. Beclin-1 and class III phosphatidylinositide 3-kinase (VPS 34))^13,14^ are synthesized and recruited to initiate the nucleation of the phagophore, and as the phagophore elongates and grows into a complete autophagosome, a protein called microtubule-associated protein 1A/1B-light chain 3B (LC3B) starts to be synthesized from its precursor LC3A and becomes localized on autophagosomes^13^. As autophagy proceeds, autophagosomes begin to fuse with lysosomes and mature into degradative autolysosomes with LC3B internalized. At the same time, other regulatory proteins (e.g. tectonin beta-propeller repeat-containing protein-1 (TECPR-1) and UV radiation resistance-associated gene protein (UVRAG))^14,15^ are synthesized and recruited to promote the maturation of autolysosomes. Eventually, the pathogens as well as LC3B are degraded in autolysosomes by the enzymes and substances delivered from lysosomes. As a result, autophagy can be considered as an innate immune response against bacterial infection and studies have shown that autophagy can restrict intracellular growth of many bacteria such as streptococcus A^16^, mycobacterium tuberculosis^17^, listeria monocytogenes^18^, and C. trachomatis^19^.

Although chlamydial infection can be easily managed by macrolides or tetracyclines, the constant recurrence, the potential to develop antibiotic resistance^20^, the safety of antibiotic use during pregnancy^21–23^ and the common systemic side effects of antibiotics^24,25^ are always problems and concerns encountered in the healthcare setting. In an effort to control the prevalence of chlamydia, various screening programs have been established in different countries around the world. However, new cases and recurrent cases still pose a challenge in disease control^2^. Therefore, a safe non-antibiotic based therapy needs to be developed to provide alternative treatment choices for physicians and patients. C. trachomatis primarily targets epithelial cells as the first step to establish genital infection and forms inclusions to escape the endo-lysosomal degradation pathway. These two conditions predispose the genital epithelial cells with higher vulnerability to C. trachomatis compared to other immune cells in the genital tract. As a result, a therapy that can reduce bacterial binding to epithelial cells and induce autophagy in infected epithelial cells for the elimination of intracellular bacteria would be beneficial for combating the infection.

The use of small interfering RNA (siRNA) as a gene therapy to combat sexually transmitted infections has gained a lot of success during the past decade^26–29^. As a result, we propose a novel combination therapy involving the application of a siRNA-polyethylenimine-encapsulated nanoparticle fabricated with poly(lactic-co-glycolic acid)-polyethylene glycol (siRNA-PEI-PLGA-PEG NP) for the knock down of PDGFR-β expression and the simultaneous induction of autophagy as a strategy to prevent/reduce sexually transmitted chlamydia infection in women. This therapy can help prevent/reduce the acquisition and recurrence of chlamydia infection when used topically in the vagina prior to sexual intercourse. Knocking down PDGFR-β will prevent/reduce chlamydia entry into target host cells and inducing autophagy by encapsulating a cationic polymer PEI will help degrade the intracellular pathogens that have already invaded as mentioned above. This topical siRNA-based therapy will work locally to minimize the systemic side effects such as those caused by oral administration of antibiotics and the use of siRNA is associated with little to no development of resistance in bacterial cells.

The application of NP in pharmaceutical sciences has gained a lot of interests during the past three decades due to its advantages in promoting drug delivery. The diverse composition of NP with polymers, lipids and other materials improves bioavailability, biocompatibility and achieves sustained or stimuli-responsive drug release profiles, targeted drug delivery, enhanced pharmacokinetics, barrier penetration and so forth^30–36^.

Among all the biomaterials, PLGA is the most attractive and extensively used polymer for the development of intravaginal NP formulations. PLGA is a FDA-approved polymer that has been widely used in the preparation of NP due to its attractive properties including biodegradability, biocompatibility and versatility for encapsulating both small molecules and large molecules, capability for sustained drug release and possibility for surface modifications^37^. PLGA NP is highly biocompatible because PLGA can naturally degrade into lactic acid and glycolytic acid via ester bond hydrolysis within the body^38^. These two monomers can either enter the tri-carboxylic acid cycle for further breakdown into carbon dioxide and water or remain unchanged, and subsequently eliminated from the body^39,40^. Woodrow *et al*. successfully achieved intravaginal gene silencing in mice using siRNA-loaded PLGA NPs^41^. The use of PLGA NP was safe *in vivo* without triggering any immune responses^41^. Currently, one PLGA-based NP product (Eligard®) has been approved by the FDA for treating prostate cancer^42^.

Even though the use of PLGA NPs is safe and effective in gene knockdown, the mucus penetration ability of PLGA NPs was largely hindered by the hydrophobic interaction between the polymers and mucin fibers. In order to improve this, Hanes *et al*. have modified the hydrophobic PLGA NP with a dense coating of low molecular weight PEG resulting in significant improvement in mucus penetration^36^. Moreover, the addition of PEG to the system also improves the stability of NP in complex physiological environments by reducing their interactions with proteins and small molecules^43^. PEG is a FDA-approved polymer and its application is safe in humans and has been used in many FDA-approved medications including intravenous injections^44,45^.

Polyethylenimine (PEI) is a cationic polymer that can effectively condense hydrophilic siRNA through electrostatic interaction and facilitate effective encapsulation of siRNA into NPs^46^. Studies have shown that the use of PEI can improve the encapsulation efficiency of siRNA from 43% to 86%^46^. Moreover, like all cationic polymers potentially, PEI is capable of inducing autophagy in mammalian cells^47^. Chia-wei *et al*. have previously reported that branched PEI (25 K) was capable of inducing autophagy in mammalian cells. However, the use of PEI as a therapeutic therapy for promoting autophagy is largely limited by its cytotoxic effects, which are largely attributed to the permeabilization of plasma membranes^48^, decrease of nuclear size, decrease of lysosomal mass/pH, the permeabilization of mitochondrial membrane^49^ and induction of apoptosis and necrosis^47^.

As a result, we developed a siRNA-PEI-PLGA-PEG NP formulation to knock down PDGFR-β and promote autophagic flux in host cells simultaneously as a defensive strategy against C. trachomatis infection. The use of NP not only efficiently delivers siRNA into target cells but also reduces the cytotoxicity of PEI without compromising its ability in promoting autophagy.

## Results

### Autophagy study by siRNA-PEI-PLGA-PEG NP

**Figure 1:**
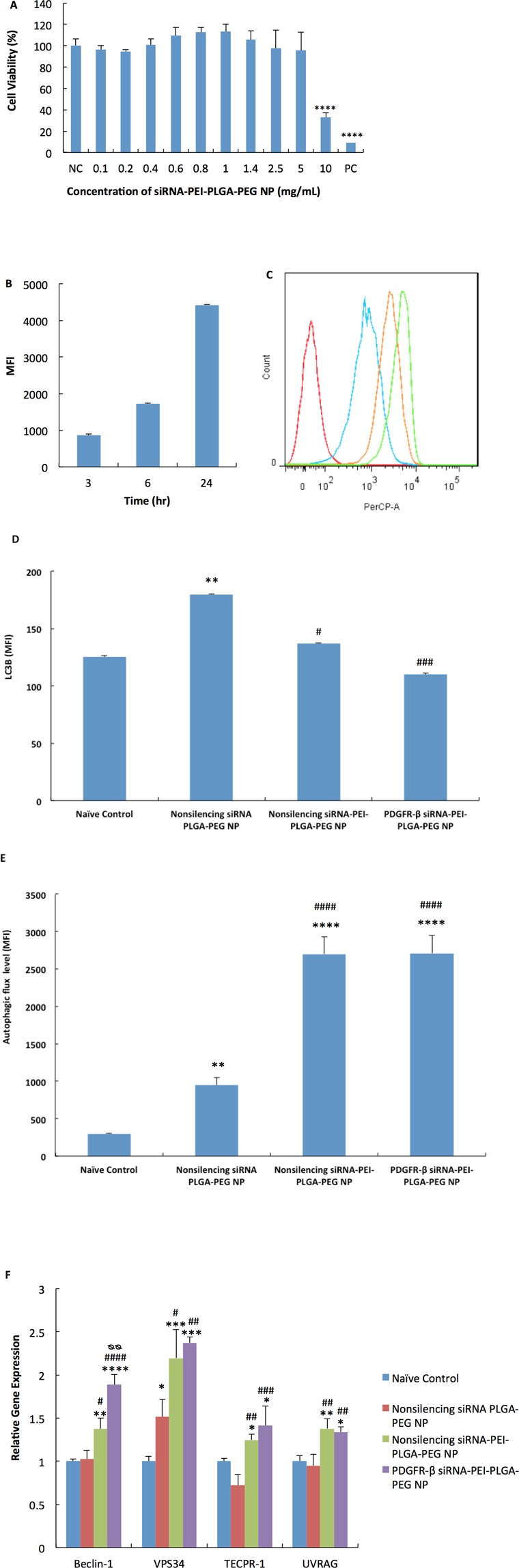
*In vitro* induction of autophagy in VK2/E6E7 cells by various NP formulations at a concentration of 1.334 mg/mL with an incubation period of 48 hr. (**A**) *In vitro* cytotoxicity of non-silencing siRNA-PEI-PLGA-PEG NP in VK2/E6E7 cells after 48 hr incubation. Results were measured by MTS assay. NC: negative control, cell culture medium, PC: positive control, 1 M acrylamide. ****p < 0.0001, compared to NC. Values represent the mean ± SD, n = 3. (**B**,**C**) *In vitro* cell uptake of Cy3-labeled siRNA-PEI-PLGA-PEG NP at a concentration of 1.334 mg/mL over a period of 24 hr. (**B**) Cumulative uptake of Cy3-labeled siRNA-PEI-PLGA-PEG NP by VK2/E6E7 cells quantified by MFI (mean fluorescence intensity) over time. (**C**) A representative histogram of uptake of Cy3-labeled siRNA-PEI-PLGA-PEG NP from n = 3. Results were quantified by flow cytometry. Red: Non-labeled siRNA-PEI-PLGA-PEG NP, blue: 3 hr, orange: 6 hr, green: 24 hr. Values represent the mean ± SD, n = 3. (**D**) Intracellular level of LC3B quantified by flow cytometry (**E**) Intracellular level of autophagic flux quantified by CYTO-ID® Autophagy detection kit with flow cytometry. (**F**) Relative gene expression of autophagy-regulatory genes quantified by qRT-PCR with GAPDH as endogenous control. Values in (**D**–**F**) represent the mean ± SD, n = 3. MFI: mean fluorescence intensity. *Compared to naïve control, *p < 0.05, **p < 0.01, ***p < 0.001, ****p < 0.0001. ^#^Compared to nonsilencing siRNA PLGA-PEG NP, ^#^p < 0.05, ^##^p < 0.01, ^###^p < 0.001, ^####^p < 0.0001. ^øø^p < 0.01 compared to nonsilencing siRNA-PEI-PLGA-PEG NP.

The PDGFR-β-siRNA-PEI-PLGA-PEG NP showed a particle size of 260.30 ± 6.43 nm and a zeta potential of −17.8 ± 5.2 mV in PBS, pH 7.4. Vaginal epithelial cells, VK2/E6E7, were capable of tolerating up to 5 mg/mL of nonsilencing siRNA-PEI-PLGA-PEG NP with an incubation time of 48 hr before any decline in cell viability was observed (Fig. 1A). Cellular uptake of siRNA was rapid and efficient and the increase in intracellular siRNA followed a time-dependent manner (Fig. 1B,C).

The number of autophagosome is a widely used marker for studying autophagy and it correlates well with the amount of LC3B, therefore, the intracellular level of LC3B was first quantified to identify changes in the dynamic pathway of autophagy. Our results indicated that compared to naïve control, nonsilencing siRNA-PLGA-PEG NP significantly increased the intracellular level of LC3B by 43.1 ± 11.9% (p = 0.0029), while nonsilencing siRNA-PEI-PLGA-PEG NP and PDGFR-β siRNA-PEI-PLGA-PEG NP did not increase the intracellular level of LC3B at all. However, this does not mean that nonsilencing siRNA-PEI-PLGA-PEG NP and PDGFR-β siRNA-PEI-PLGA-PEG NP did not exert an effect on the formation and conversion of autophagosomes, because when we compared the LC3B levels in these two groups to that in nonsilencing siRNA PLGA-PEG NP-treated group, we found that the intracellular levels of LC3B were significantly decreased by 23.8 ± 4.5% (p = 0.0119) and 38.7 ± 5.5% (p = 0.0006) respectively, and no significant change in LC3B was detected between nonsilencing siRNA-PEI-PLGA-PEG NP-treated group and PDGFR-β siRNA-PEI-PLGA-PEG NP-treated group (Fig. 1D).

Autophagosome is an intermediate structure in the autophagic flow when it is consistently formed and converted to autolysosome, but its number detected at any specific time does not represent the autophagic degradation activity in cells^50^. Therefore, in order to investigate whether PEI encapsulation into NP would promote autophagic degradation activity in VK2/E6E7 cells, a widely accepted autophagy detection kit (CYTO-ID® Autophagy detection kit) was used subsequently to quantify the level of autophagic flux (defined as a measure of autophagic degradation activity) in cells. The results revealed that NP without the encapsulation of PEI only increased the intracellular autophagic flux by about 3 folds (p = 0.0073) compared to naïve control (Fig. 1E). In contrast, PEI-encapsulated NP significantly enhanced the autophagic flux in cells by about 9 folds (p < 0.0001 was given by GraphPad Prism 6 for the actual p value) compared to naïve control, regardless if it was nonsilencing siRNA or PDGFR-β siRNA encapsulated in the NP (Fig. 1E). Therefore, the NP formulation containing PEI could enhance the autophagic degradation activity in VK2/E6E7 cells a lot more than that without PEI, even though all three NP formulations could promote autophagic degradation activity.

To further investigate how the autophagic stages are influenced, we looked at changes in the gene expression of four common autophagy regulatory genes: Beclin-1, VPS34, UVRAG and TECPR-1. Beclin-1 and VPS34 take part in the vacuolar sorting and autophagosome biosynthesis^13,14^, thus are markers of the initiation stage of autophagy while UVRAG and TECPR-1 are required in autophagosome-lysosome fusion^14,15^, which are markers of the degradation stage. Our results showed that nonsilencing siRNA PLGA-PEG NP only caused a significant increase in the expression of VPS34 (p = 0.0498) compared to naïve control. In contrast, both nonsilencing siRNA-PEI-PLGA-PEG NP and PDGFR-β siRNA-PEI-PLGA-PEG NP significantly increased the expression of all four genes (p values are shown in Table S1). Knocking down PDGFR-β also significantly further enhanced the expression of Beclin-1 (p = 0.0011) compared to nonsilencing siRNA-PEI-PLGA-PEG NP (Fig. 1F).

### Autophagy study by free PEI

**Figure 2:**
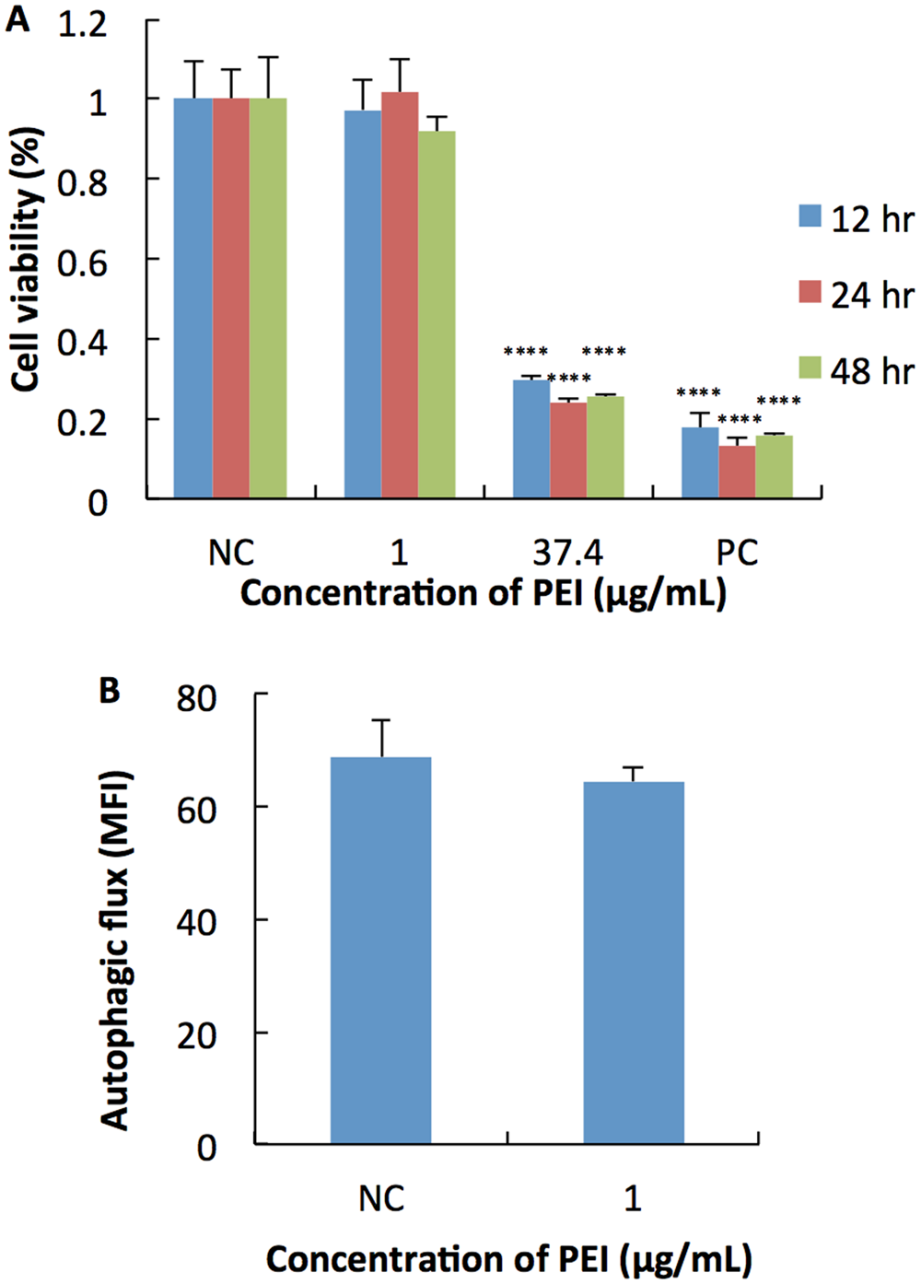
(**A**) *In vitro* cytotoxicity of free PEI in VK2/E6E7 cells after 12, 24 and 48 hr incubation. Results were measured by MTS assay. NC: negative control, cell culture medium, PC: positive control, 1 M acrylamide, ****p < 0.0001, compared to NC. (**B**) The level of autophagic flux quantified by CYTO-ID^®^ Autophagy detection kit with flow cytometry. NC: negative control, cell culture medium. MFI: mean fluorescence intensity. Values represent the mean ± SD, n = 3.

Based on the results, siRNA-PEI-PLGA-PEG NP containing PEI appeared to play an important role in increasing the autophagic degradation activity in VK2/E6E7 cells and promoting both the initiation and degradation stages of autophagy. To better understand the mechanism of action, we next examined whether free PEI could enhance autophagic degradation activity as the PEI-containing formulations did. We found that only VK2/E6E7 cells treated with 1 µg/mL showed healthy morphology while cells treated with other concentrations (>1 µg/mL) showed significant cell lysis compared to naïve control (data not shown) under microscope, rendering them ineligible for downstream studies. As a result, only cells treated with 1 µg/mL or naïve control were processed for Cyto-ID staining. At the same time, in order to confirm the results seen under the microscope, MTS cell viability assay was conducted using two selected concentrations: 1 µg/mL and 37.4 µg/mL. The concentration of 37.4 µg/mL of PEI was equivalent to the concentration of PEI in siRNA-PEI-PLGA-PEG NP when dosed at a concentration of 1.334 mg/mL, and the results for MTS matched what was observed under the microscope: 1 µg/mL was non-cytotoxic while 37.4 µg/mL caused massive cell death (Fig. 2A). When we looked at the autophagic degradation activity, we found that 1 µg/mL of free PEI did not increase autophagic degradation activity in VK2/E6E7 cells at all compared to naïve control (Fig. 2B). As a result, free PEI could not induce autophagy within its noncytotoxic concentration range and the encapsulation of PEI into PLGA-PEG NP helped reduce its cytotoxicity, thereby making it possible to exert its role in inducing autophagy.

### Downregulation of PDGFR-β in VK2/E6E7 cells

**Figure 3:**
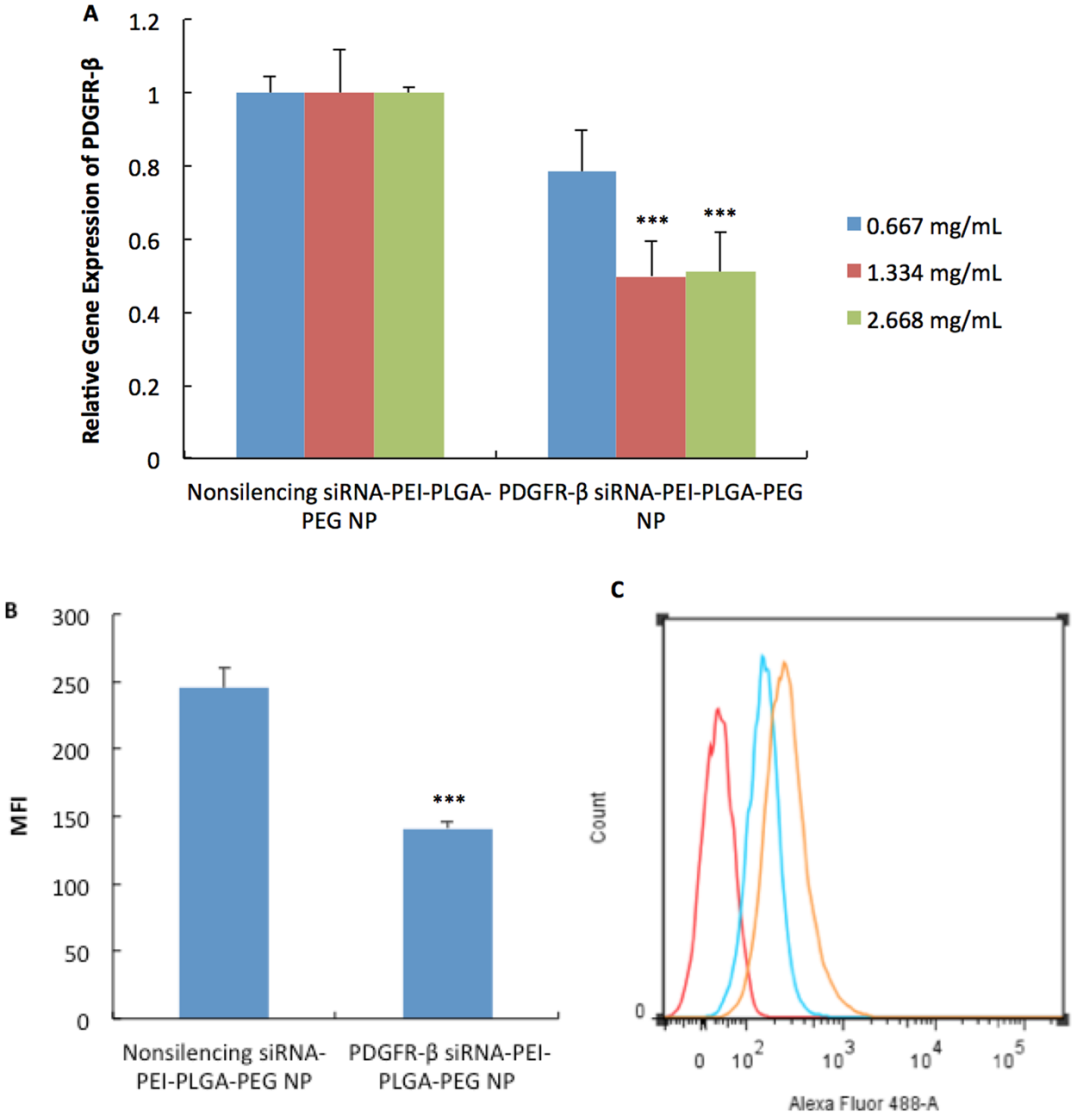
(**A**) *In vitro* mRNA downregulation of PDGFR-β in VK2/E6E7 cells by PDGFR-β siRNA-PEI-PLGA-PEG NP at different concentrations after 48 hr incubation. Results were measured by qRT-PCR. GAPDH was used as endogenous control. ***p < 0.001 compared to nonsilencing siRNA-PEI-PLGA-PEG NP. Values represent the mean ± SD, n = 3. (**B**,**C**) *In vitro* protein downregulation of PDGFR-β in VK2/E6E7 cells by PDGFR-β siRNA-PEI-PLGA-PEG NP at a concentration of 1.334 mg/mL after 48 hr incubation. Results were measured by flow cytometry. (**B**) PDGFR-β protein downregulation quantified by MFI (mean fluorescence intensity). Values represent the mean ± SD, n = 3. ***p < 0.001 compared to nonsilencing siRNA-PEI-PLGA-PEG NP. (**C**) A representative histogram from n = 3. Red: isotype control, blue: PDGFR-β siRNA-PEI-PLGA-PEG NP, orange: nonsilencing siRNA-PEI-PLGA-PEG NP.

**Figure 4:**
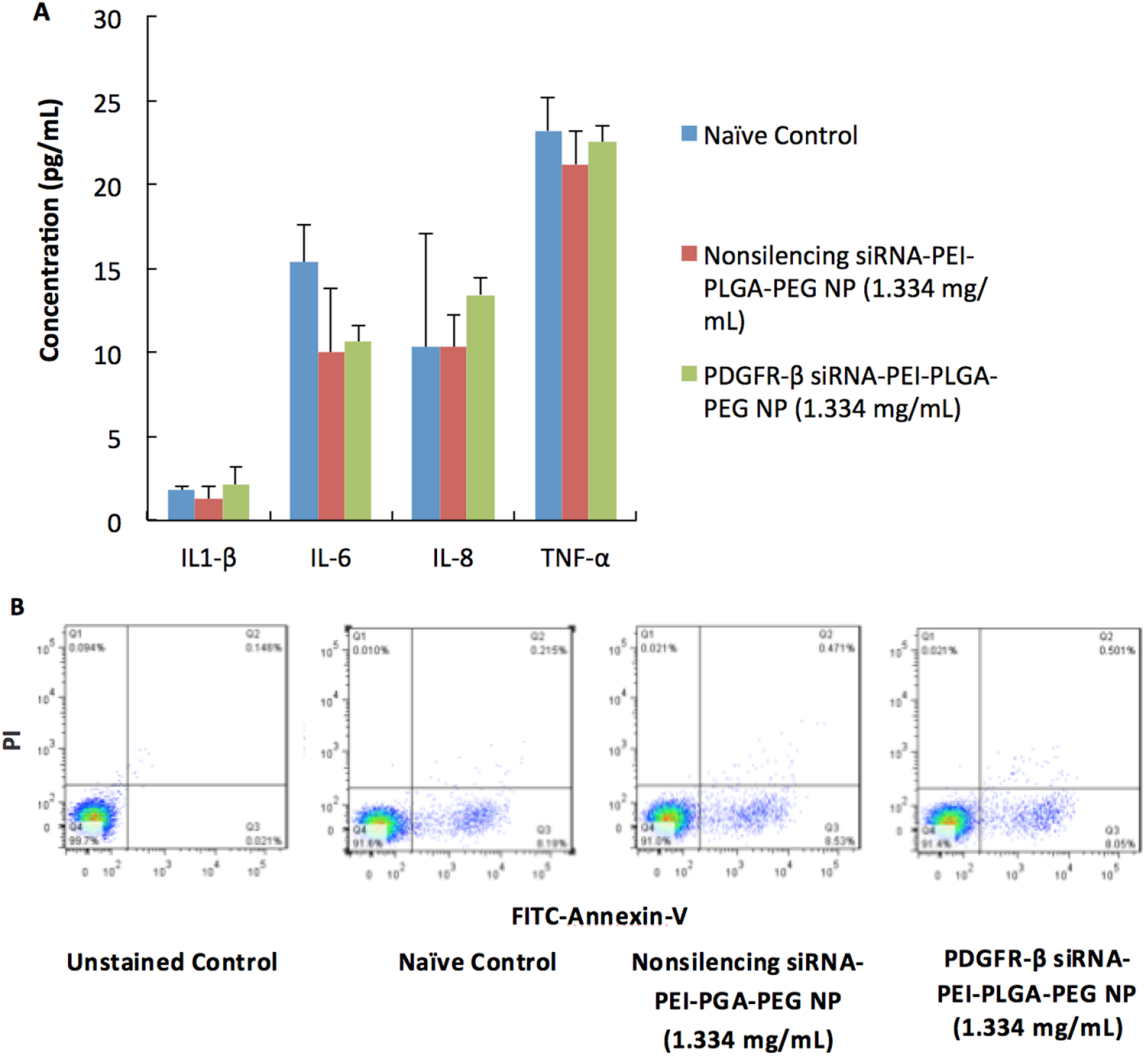
(**A**) *In vitro* proinflammatory cytokine production in VK2/E6E7 cells when cells were treated with PBS (naïve control), nonsilencing siRNA-PEI-PLGA-PEG NP (1.334 mg/mL) or PDGFR-β siRNA-PEI-PLGA-PEG NP (1.334 mg/mL) for 48 hr. Values represent the mean ± SD, n = 3. (**B**) Representative flow cytometry plots of apoptosis measured by FITC Annexin V/Dead Cell Apoptosis Kit. PI: Propidium iodide. Data was collected when VK2/E6E7 cells were treated with PBS (naïve control), nonsilencing siRNA-PEI-PLGA-PEG NP (1.334 mg/mL) or PDGFR-β siRNA-PEI-PLGA-PEG NP (1.334 mg/mL) for 48 hr. Experiments were conducted in triplicate.

In order to achieve gene knockdown of PDGFR-β, the sequence of PDGFR-β siRNA, which has been widely used in previous published literatures^5,51^ was selected and evaluated on VK2/E6E7 cells. Firstly, a dose-dependent study was performed to evaluate the downregulation of PDGFR-β mRNA in VK2/E6E7 cells. Results showed that PDGFR-β siRNA-PEI-PLGA-PEG NP (1.334 mg/mL) can significantly reduce PDGFR-β mRNA in VK2/E6E7 cells by 50% (p = 0.0002) compared to nonsilencing siRNA-PEI-PLGA-PEG NP. Doubling the siRNA concentration to 2.668 mg/mL did not further decrease gene knockdown (Fig. 3A), indicating that the maximum knockdown efficiency of this siRNA sequence was 50%. We also compared our mRNA downregulation results with the papers (mentioned above) that used this sequence as well (since only mRNA downregulation data was reported in these papers), and they also reported a maximum knockdown of about 50%^5,51^. As a result, the concentration of 1.334 mg/mL was selected for the evaluation of protein downregulation. At the concentration of 1.334 mg/mL, PDGFR-β siRNA-PEI-PLGA-PEG NP can significantly reduce PDGFR-β protein expression by 43% (p = 0.0003 with two-sided, unpaired T test) (Fig. 3B,C). It was also confirmed that at this concentration, neither nonsilencing siRNA-PEI-PLGA-PEG NP or PDGFR-β siRNA-PEI-PLGA-PEG NP had a significant impact on pro-inflammatory cytokine production (IL-1β, IL-6, IL-8 and TNF-α) (Fig. 4A) or induction of apoptosis (Fig. 4B) in VK2/E6E7 cells compared to naïve control treated with growth medium. The dose of 1.334 mg/mL would be the optimal dose for conducting downstream studies.

### Intracellular production of C. trachomatis RB in VK2/E6E7 cells and extracellular release of C. trachomatis in cell culture medium

**Figure 5:**
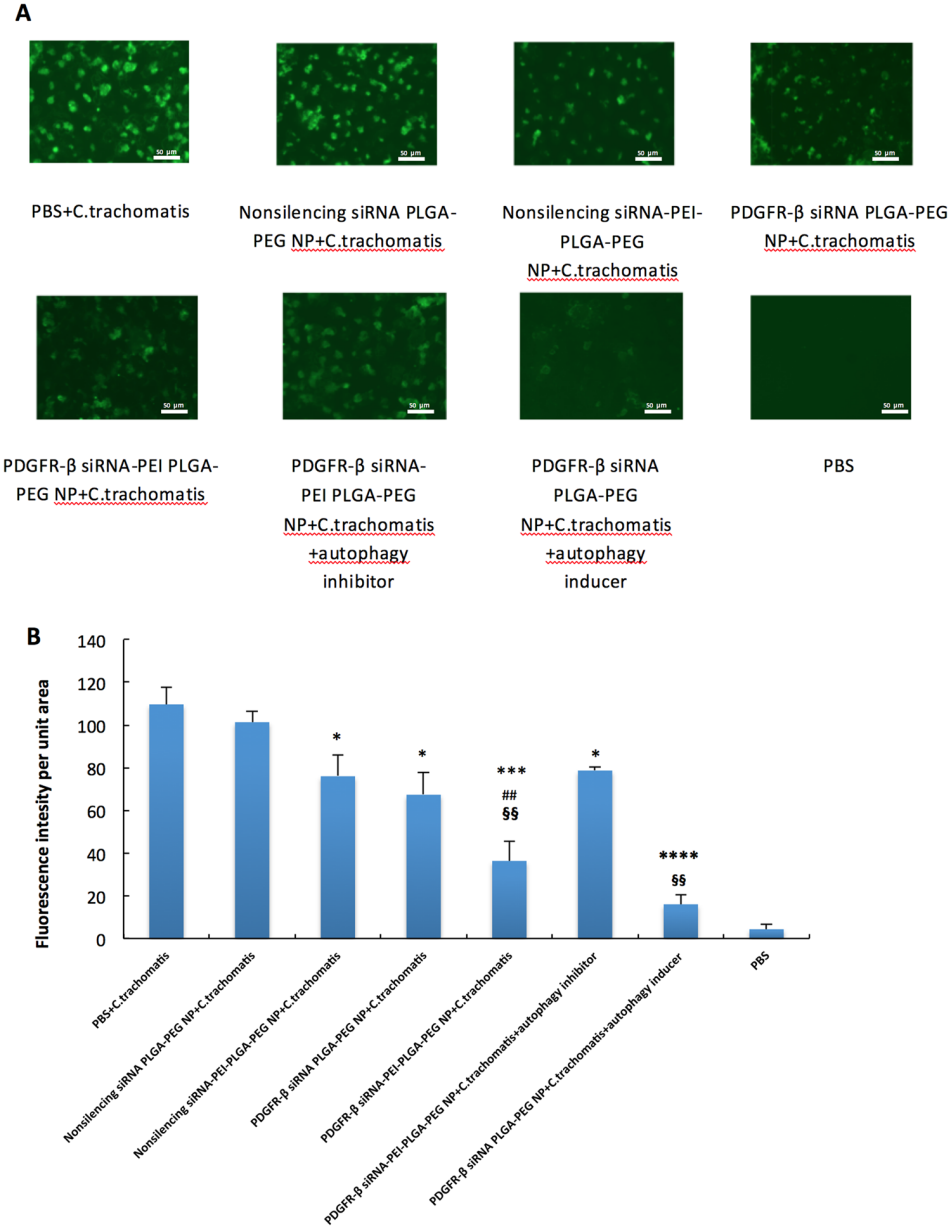
Intracellular production of C. trachomatis RBs in VK2/E6E7 cells. Cells were incubated with PBS, non-silencing siRNA PLGA-PEG NP (1.334 mg/mL), non-silencing siRNA-PEI-PLGA-PEG NP (1.334 mg/mL), PDGFR-β siRNA PLGA-PEG NP (1.334 mg/mL) or PDGFR-β siRNA-PEI-PLGA-PEG NP (1.334 mg/mL), PDGFR-β siRNA-PEI-PLGA-PEG NP (1.334 mg/mL) and autophagy inhibitor (bafilomycin A, 50 mM), PDGFR-β siRNA PLGA-PEG NP (1.334 mg/mL) and autophagy inducer (rapamycin, 100 nM) for 48 hr and then infected with C. trachomatis. Images were taken 24 hr post C. trachomatis infection. (**A**) Intracellular C. trachomatis RB foci (green fluorescence) were visualized using fluorescence microscopy. Experiments were conducted n = 3 and a group of representative images were shown. (**B**) Semi-quantitative measurements of intracellular C. trachomatis RB foci were accomplished using Image J software. Values represent the mean ± SD, n = 3. *p < 0.05, ***p < 0.001, ****p < 0.0001 compared to non-silencing siRNA PLGA-PEG NP + C.trachomatis, ^##^p < 0.01 compared to nonsilencing siRNA-PEI-PLGA-PEG NP + C. trachomatis, ^§§^p < 0.01 compared to PDGFR-β siRNA-PEI-PLGA-PEG NP + C. trachomatis.

**Figure 6:**
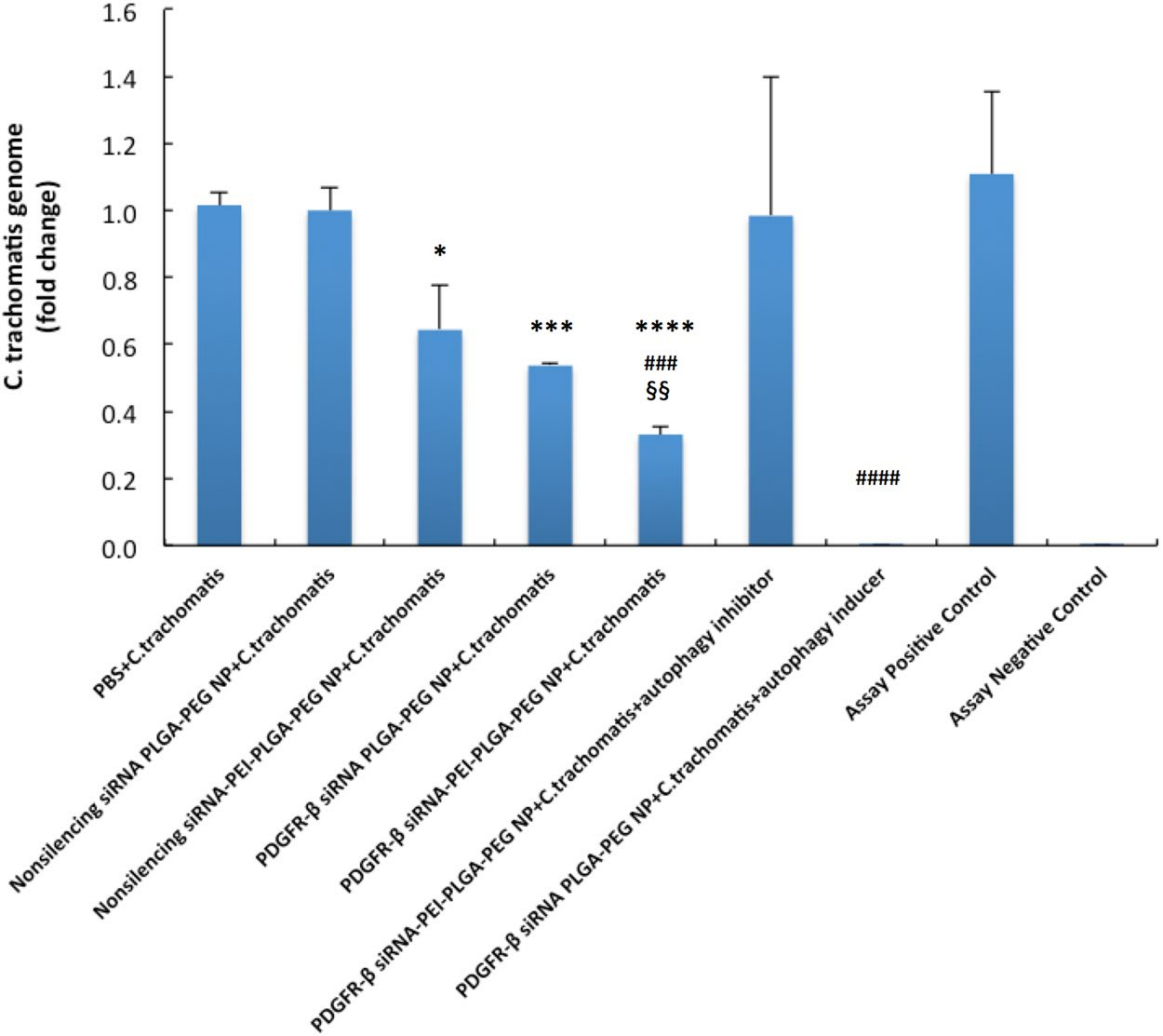
*In vitro* quantitation of C. trachomatis genomes in the supernatant of VK2/E6E7 cell culture. Cells were incubated with PBS, non-silencing siRNA PLGA-PEG NP (1.334 mg/mL), non-silencing siRNA-PEI-PLGA-PEG NP (1.334 mg/mL), PDGFR-β siRNA PLGA-PEG NP (1.334 mg/mL) or PDGFR-β siRNA-PEI-PLGA-PEG NP (1.334 mg/mL), PDGFR-β siRNA-PEI-PLGA-PEG NP (1.334 mg/mL) and autophagy inhibitor (bafilomycin A, 50 mM), PDGFR-β siRNA PLGA-PEG NP (1.334 mg/mL) and autophagy inducer (rapamycin, 100 nM) for 48 hr and then infected with C. trachomatis. Supernatant containing newly produced C. trachomatis genomes was collected 24 hr post C. trachomatis infection and quantified by qRT-PCR. Values represent the mean ± SD, n = 3. *p < 0.05, ***p < 0.001, ****p < 0.0001, compared to non-silencing siRNA PLGA-PEG NP + C.trachomatis, ^###^p < 0.001, ^####^p < 0.0001 compared to nonsilencing siRNA-PEI-PLGA-PEG NP + C. trachomatis, ^§§^p < 0.01 compared to PDGFR-β siRNA-PEI-PLGA-PEG NP + C. trachomatis. Sample of assay positive control and negative control was provided by the kit manufacturer and results of both met quality control criterion required in the manual.

24-hr after infection of C. trachomatis EBs, non-silencing siRNA-PEI-PLGA-PEG NP treatment that could promote the autophagic degradation pathway, significantly decreased the intracellular RBs and extracellular C. trachomatis by approximately 25% (p = 0.0426) (Fig. 5) and 36% (p = 0.0460) (Fig. 6), respectively. PDGFR-β siRNA PLGA-PEG NP treatment that could reduce the binding of C. trachomatis significantly decreased the intracellular RBs and extracellular C. trachomatis by approximately 34% (p = 0.0316) (Fig. 5) and 46% (p = 0.0007) (Fig. 6), respectively. When autophagic degradation pathway was promoted simultaneously as C. trachomatis binding was inhibited, the intracellular production of RBs was decreased by about 65% (p = 0.0008) (Fig. 5) and the extracellular release of C. trachomatis was reduced by about 67% (p = 0.00005) (Fig. 6). When autophagy inducer was introduced in the PDGFR-β siRNA PLGA-PEG NP group, the intracellular production of RBs and extracellular release of C. trachomatis was decreased down to the lowest level, whereas, when autophagy inhibitor was introduced in the non-silencing siRNA-PEI-PLGA-PEG NP group, the protective effect was reversed. Overall, knocking down PDGFR-β in combination with augmenting autophagy pathway worked simultaneously to reduce the C. trachomatis infection in VK2/E6E7.

## Discussion

Chlamydia infection is transmitted through unprotected sexual intercourse and can infect the vagina and cervix^52,53^. Even though the organism is susceptible to antimicrobial agents, the occurrence of multi-drug resistant strains poses big challenges in combating this pathogen. Considering the fact that an effective vaccine is still not available, we believe the use of a non-antibiotic based microbicide is an excellent alternative strategy to help prevent/reduce chlamydia infection in women in addition to regular screening of the pathogen in susceptible populations^1^.

In the design of our microbicide, we combined two preventive strategies together, which includes the reduction of bacterial entry into host cells and augmentation of host defense against C. trachomatis. The strategies take advantage of the special characteristics of siRNA-PEI-PLGA-PEG NP, in which PEI plays an important role in promoting the autophagy in host cells as well as improving the encapsulation of siRNA into NP^46^. NP facilitates the delivery of siRNA for sufficient PDGFR-β gene knockdown and also helps reduce the cytotoxicity of PEI, thus making it possible to deliver adequate amounts of PEI into cells for inducing autophagy without causing cytotoxicity. Our findings are consistent with what has been reported whereby free PEI is highly toxic to cell lines e.g. in Chia-wei’s study^47^, the cells can only tolerate 4 µg/mL of PEI for 4 hr and 1 µg/mL PEI for 48 hr. When encapsulated into NP, the toxicity of PEI was greatly minimized, based on the results of the MTS assay. VK2/E6E7 cells can tolerate up to 139.5 mg/mL of PEI (equivalent to 5 mg/mL siRNA-PEI-PLGA-PEG NP) for 48 hr without decreasing cell viability. More importantly, the NP can deliver sufficient amounts of PEI into cells to induce autophagy without causing inflammation, apoptosis or any decrease in cell viability. By using a NP formulation, we successfully resolved the problem associated with the cytotoxic effects of PEI but still retained the beneficial properties of PEI in inducing autophagy. We believe the key to decreasing the cytotoxicity of PEI is through encapsulation with NP, which prevents the direct contact between the cationic polymer and membranes of cells and organelles.

Since autophagy is a dynamic process, the induction of autophagy was evaluated using three different methods. As mentioned in the introduction, intracellular LC3B correlates well with the number of autophagosomes, which would further indicate changes occurring during the initiation and degradation stages of autophagy since autophagosomes are formed during the initiation stage and degraded during the degradation stage. Generally, an increase in LC3B (equal increase in the amount of autophagosome) can indicate: (1) an increased initiation stage followed by an unchanged degradation stage or (2) an unchanged initiation stage followed by a decreased degradation stage or (3) an increased initiation stage followed by a decreased degradation stage or (4) a predominant increased initiation stage followed by an increased degradation stage causing a combined effect of increased LC3B or (5) a decreased initiation stage followed by a predominant decreased degradation stage causing a combined effect of increased LC3B (Table S2). These conditions also correspond to different changes in autophagic flux, known as the autophagic degradation activity (Table S2). Possible reasons for a decrease or no change in the levels of LC3B are also listed in Table S2.

To summarize the results from all three studies, with respect to nonsilencing siRNA PLGA-PEG NP, there was an observed increase in the level of autophagic flux, an increase in the level of LC3B and an increase in the gene expression of VPS34 compared to naïve control. It appears that the results collected for the nonsilencing siRNA PLGA-PEG NP group possibly fits scenario No.4 in Table S2 except for a lack in the upregulation of the late-stage autophagy regulatory genes. This could be explained by the possibility that other autophagy regulatory genes/proteins may be involved, leading to the promotion of the degradation stage of autophagy. Therefore, the nonsilencing siRNA PLGA-PEG NP augmented autophagic flux by potentially promoting the initiation and degradation stages simultaneously, but the degradation stage was not promoted as much as the initiation stage, putting a limit on the promotion of autophagic flux.

However, when PEI was encapsulated into NP, we found that compared to naïve control, nonsilencing siRNA-PEI-PLGA-PEG NP and PDGFR-β siRNA-PEI-PLGA-PEG NP did not change intracellular LC3B but significantly increased autophagic flux and upregulated all four autophagy-related genes (scenario No.6, Table S2). This was because the increased initiation stage was followed by an equally increased degradation stage, causing the increased number of autophagosomes to be completely converted and degraded in the degradation stage. Therefore, the encapsulation of PEI into NP made a significant contribution to promoting the degradation stage of autophagy, therefore producing a greater increase in autophagic flux than NP without PEI.

Subsequently the comparisons were made between nonsilencing siRNA PLGA-PEG NP and nonsilencing siRNA-PEI PLGA-PEG NP. We found that in comparison to nonsilencing siRNA PLGA-PEG NP containing no PEI, the encapsulation of PEI into PLGA-PEG NP (regardless it is nonsilencing siRNA or siRNA PDGFR-β encapsulated) significantly decreased intracellular LC3B, increased autophagic flux, and augmented the expression of four autophagy-regulatory genes. Based on the analysis, the profiles of nonsilencing siRNA-PEI-PLGA-PEG NP and PDGFR-β siRNA-PEI-PLGA-PEG NP are likely to fit the criteria described in scenario No.11, Table S2. Even though we did not observe higher increase in the gene expression of TECPR-1 and UVRAG than Beclin-1 and VPS 34, scenario No.11 was the only one meeting all the other conditions. We thought there might be some other genes/proteins involved in the pathway of autophagy that were not measured in this study (like mentioned above) and all the participating factors led to the changes of LC3B and autophagic flux observed in this study. Therefore, based on the comparison, we believed that, on the basis of nonsilencing siRNA PLGA-PEG NP, PEI encapsulation could further promote the initiation stage and degradation stage simultaneously and more promotion could possibly occur in the degradation stage.

Previous literature has shown that free PEI can enhance the formation of autophagosomes and LC3B^47,49^ mainly during the early stages of autophagy. Whether or not the late stages of autophagy is affected (e.g., fusion of autophagosomes and lysosomes) is not clear. This made it difficult to conclude that the autophagic flux was augmented since as discussed earlier, blocking the late stage would halt the progression of autophagy, rendering the targets for degradation to accumulate in autophagosomes instead of being degraded in autolysosome. In our study, we have provided evidence showing that our nonsilencing siRNA-PEI-PLGA-PEG NP and PDGFR-β siRNA-PEI-PLGA-PEG NP can promote autophagic flux (degradative activity of autophagy) by promoting both the formation of autophagosomes (initiation stage) and the fusion of autophagosomes with lysosomes (degradation stage) simultaneously with more promotion made in the degradation stage, which has never been reported previously. And we found that the promotion of autophagic flux is related to the upregulation of four autophagy regulatory genes (Beclin-1, VPS34, TECPR-1 and UVRAG). We are not sure what other genes/protein are also involved in this process, therefore, further studies are required to discover the roles of other possible autophagy regulated genes/proteins to provide a complete explanation for the current findings.

Xiaoling Gao *et al*. have shown that free PEI was taken up by cells via clathrin-mediated endocytosis (CME) pathway and the CME pathway plays an important role in promoting the formation of autophagosomes^49^. Previous literature has also reported that PLGA-PEG NP (~300 nm, negatively charged) can be taken up by cells through both CME, and clathrin-/caveolae-independent pathways depending on the cell type^54^. Although the mechanism of entry into vaginal epithelial cells of our siRNA-PEI-PLGA-PEG NP has not been investigated, it is possible that our siRNA-PEI-PLGA-PEG NP is also taken up by cells via the CME pathway due to the presence of PLGA-PEG which in turn promotes the formation of autophagosomes. Therefore, besides the upregulation of autophagy-regulatory genes, the cell uptake pathway of NP may also contribute to the promotion of initiation stage of autophagy.

The use of PDGFR-β siRNA-PEI-PLGA-PEG NP should start days before sexual interactions as there is a delayed onset of action of gene silencing by PDGFR-β siRNA and its use may continue as long-term since the gene knockdown effect of siRNA is transient and continuous intracellular level of siRNA may need to be maintained for sustained gene knockdown. However, the dosing frequency and duration of administration have to be determined by future *in vivo* and clinical studies as they are highly related to the formulation’s efficacy and safety profiles. Currently the prevention efficiency of our PDGFR-β siRNA-PEI-PLGA-PEG NP can not achieve 100% *in vitro* so in the future other potential receptors can be targeted simultaneously to enhance the prevention efficacy. For example, epithelial membrane protein 2 (EMP2) has been found as a potential target to reduce C. trachomatis infection and the use of genetic silencing technique or neutralizing antibody can reduce chlamydial infection *in vitro* and *in vivo*^55^. Therefore, siRNA targeting EMP2 can be co-encapsulated in our NP system and/or anti-EMP2 antibody can be conjugated to the surface our NP due to the versatility of NP drug delivery system.

## Methods

### Cell culture and C. trachomatis propagation

Vaginal epithelial cells (VK2/E6E7), C. trachomatis strain K, and McCoy cells were purchased from ATCC (Virginia, USA). Keratinocyte-SFM and its supplements were purchased from Invitrogen (Ontario, Canada). Calcium chloride was purchased from Sigma-Aldrich (Ontario, Canada). VK2/E6E7 cells were maintained at 37 °C and 5% CO_2_ with Keratinocyte-SFM medium supplemented with 0.1 ng/mL human recombinant epidermal growth factor (EGF), 0.05 mg/mL bovine pituitary extract, 44.1 mg/L calcium chloride and 100 μg/mL penicillin-streptomycin (Thermo Fisher, Ontario, Canada) (100 U/mL). McCoy cells were maintained at 37 °C and 5% CO_2_ with Eagle’s Minimum Essential Medium (Lonza, New Jersey, USA) supplemented with 10% FBS (PAA, Ontario, Canada). C. trachomatis strain K was propagated in McCoy cells according to manufacturer’s instructions and C. trachomatis EBs were harvested from infected cells by sonication in PBS (Lonza, New Jersey, USA) for 20 s and the mixer was centrifuged at 500 × g, 4 °C for 15 min. The pellet was resuspended in PBS and sonicated for another 20 s, followed by centrifugation at 30,000 × g, 4 °C for 60 min^56^.

### Preparation of NP

Nonsilencing siRNA and siRNA targeting PDGFR-β were synthesized by Dharmacon, Ontario, Canada. Cy3-labeled nonsilencing siRNA was purchased from Thermo Fisher, Ontario, Canada. Sequences are listed in supplementary information. Briefly, siRNA was first condensed by PEI and then encapsulated into NP made from the biodegradable di-block copolymer, PLGA-PEG (50/50)(10 kDa)-(2 kDa), (COOH-terminated, Advanced Polymer Materials, Quebec, Canada) using the double-emulsion evaporation method^27,57^. Equal volumes of siRNA (100 µg) and PEI (Branched PEI 25 kDa, Sigma-Aldrich, Ontario, Canada) dissolved in TE buffer, pH 7.5 were combined together at N/P ratio = 5:1. siRNA-PEI complex was then continuously emulsified with 600 µL of PLGA-PEG (20 mg/mL) dissolved in methylene chloride (Thermo Fisher, Ontario, Canada) for 15 s. The primary emulsion was further emulsified with 4.3 mL of 2% polyvinyl alcohol (PVA, 31 ~ 50 kDa, Sigma-Aldrich, Ontario, Canada) for 2-3 min, forming a w/o/w secondary emulsion. The secondary emulsion was stirred at 4 °C for more than 3 hr to evaporate the organic solvent and harden the NP. The NPs were then collected by centrifugation (20,000 × g for 15 min at 4 °C) and washed twice with water to eliminate excess PVA and unencapsulated siRNA. Nonsilencing siRNA NP (containing no PEI), nonsilencing siRNA-PEI-PLGA-PEG NP, cy3-labeled siRNA-PEI-PLGA-PEG NP and PDGFR-β siRNA–PEI NP were prepared using this method.

### *In vitro* studies of autophagy induction

LC3B level was determined by immunofluorescence. Briefly, 0.7 × 10^5^ VK2/E6E7 cells were seeded onto 24-well plate with 500 µL growth medium and maintained overnight. On the day of the experiment, nonsilencing siRNA NP (containing no PEI), nonsilencing siRNA-PEI-PLGA-PEG NP and PDGFR-β siRNA–PEI PLGA-PEG NP were used to treat cells at a concentration of 1.334 mg/mL. Cells treated with culture medium were used as naïve control. All groups were incubated at 37 °C and 5% CO_2_ for 48 hr. At the end of incubation, cells were trypsinized, washed and fixed with 2% paraformaldehyde (BD Biosciences, Ontario, Canada). Cells were permeabilized with 0.1% saponin/PBS and fixed with Fc blocker (BD Biosciences, Ontario, Canada). After that, the cells were stained with mouse anti-human anti-LC3B antibody [5H12] (Abcam, Massachusetts, USA) (1:200 dilution) and donkey anti-mouse IgG H&L (Alexa Fluor® 488) (Abcam, Cambridge, USA) (1:2000) for 30 min each on ice. Mouse monoclonal IgG1 (Abcam, Massachusetts, USA) was used as isotype control. The samples were then analyzed by flow cytometry (BD FACSCanto™ II system).

Autophagic flux was determined using the CYTO-ID® Autophagy detection kit (Enzo Life Sciences, New York, USA) with the same dosing regimen mentioned above. Cells were treated and stained according to manufacturer’s instructions and the level of autophagic flux was determined by flow cytometry.

Beclin-1, VPS 34, TECPR-1 and UVRAG gene expression were evaluated in cells treated with the same dosing regimen mentioned above. Total RNA was extracted using E.Z.N.A.® Total RNA Kit I (Omega Biotek, Georgia, USA). cDNA synthesis was performed with qScript™ cDNA SuperMix (Quanta Biosciences, Massachusetts, USA) according to the manual. qRT-PCR was performed with PerfeCTa SYBR Green SuperMix from (Quanta Biosciences, Massachusetts, USA) on QuantStudio™ 6 Flex Real-Time PCR System (Thermo Fisher, Ontario, Canada). The thermal cycle was conducted by incubating at 95 °C for 3 min, followed by PCR amplification of 50 cycles at 95 °C for 15 s, 59 °C for 45 s. melt curve was run at 95 °C for 15 s followed by 60 °C for 1 min. GAPDH was used as an endogenous control. All primer sequences (Dharmacon, Ontario, Canada) and siRNA sequences are listed in Table S3.

### Cell uptake of Cy3-labeled siRNA-PEI-PLGA-PEG NP

0.7 × 10^5^ VK2/E6E7 cells were seeded in 24-well plates with 500 µL growth medium and maintained overnight. On the day of the experiment, cells were treated with cy3-labeled siRNA-(1.334 mg/mL) at 37 °C, 5% CO_2_ for different time intervals. At the end of incubation, cells were washed three times with PBS and analyzed by flow cytometry using the PerCP filter set.

### *In vitro* cytotoxicity study

VK2/E6E7 cells (2.5 × 10^4^) were seeded onto 96-well plates and dosed with different concentrations of nonsilencing siRNA-PEI-PLGA-PEG NP the next day. Cells were incubated at 37 °C, 5% CO_2_ for 48 hr. Treatments with growth medium and 1 M acrylamide were used as negative control and positive control, respectively. Cell viability was measured using the CellTiter 96® Aqueous One Solution Cell Proliferation Assay (Promega, Wisconsin, USA).

0.7 × 10^5^ VK2/E6E7 cells were seeded onto 24-well plates. The next day, cells were treated with nonsilencing siRNA-PEI-PLGA-PEG NP or PDGFR-β siRNA–PEI PLGA-PEG NP at a concentration of 1.334 mg/mL and incubated at 37 °C, 5% CO_2_ for 48 hr. Cells were then centrifuged at 20,000 × g, 4 °C for 15 min to remove NP. The supernatant was analyzed by ELISA to quantify the concentration of pro-inflammatory cytokines (IL-1β, IL-6, IL-8 and TNF-α ELISA kits were purchased from R&D systems, Minnesota, USA). Cells were then trypsinized, stained with FITC Annexin V/Dead Cell Apoptosis Kit (Thermo Fisher, Ontario, Canada) and analyzed for apoptosis using flow cytometry.

### *In vitro* PDGFR-β downregulation study

0.7 × 10^5^ VK2/E6E7 cells were seeded onto 24-well plates and incubated overnight. Cells were treated with either growth medium, nonsilencing siRNA-PEI-PLGA-PEG NP, or PDGFR-β siRNA-PEI-PLGA-PEG NP and incubated at 37 °C, 5% CO_2_ for 48 hr. At the end of incubation, cells were washed three times with growth medium and RNA was extracted using E.Z.N.A.^®^ Total RNA Kit I. cDNA synthesis was performed using qScript™ cDNA SuperMix and qRT-PCR was performed using PerfeCTa SYBR Green SuperMix as mentioned above. The thermal cycle consisted of 95 °C for 3 min, followed by PCR amplification of 50 cycles at 95 °C for 15 s, and 59 °C for 45 s. Melt curves were run at 95 °C for 15 s followed by 60 °C for 1 min. GAPDH was used as an endogenous control.

The protein level of PDGFR-β was measured by flow cytometry. 2.8 × 10^5^ VK2/E6E7 cells were seeded onto 6-well plates, treated with formulations mentioned above and incubated at 37 °C, 5% CO_2_ for 48 hr. At the end of the study, cells were washed three times with PBS and trypsinized. Cells were fixed with 2% paraformaldehyde, blocked with 10% FBS and stained with FITC-rabbit anti-PDGFR-β antibody (958) (Santa Cruz Biotechnology, Texas, USA). The resulting samples were analyzed by flow cytometry using the AlexaFluor488 filter set.

### Chlamydia infection study

VK2/E6E7 cells (0.7 × 10^5^) were seeded onto 24-well plates and allowed to incubate overnight. Cells were treated with PBS, non-silencing siRNA PLGA-PEG NP (1.334 mg/mL), non-silencing siRNA-PEI-PLGA-PEG NP (1.334 mg/mL), PDGFR-β siRNA PLGA-PEG NP (1.334 mg/mL) or PDGFR-β siRNA-PEI-PLGA-PEG NP (1.334 mg/mL), PDGFR-β siRNA-PEI-PLGA-PEG NP (1.334 mg/mL) and autophagy inhibitor (bafilomycin A, 50 mM), PDGFR-β siRNA PLGA-PEG NP (1.334 mg/mL) and autophagy inducer (rapamycin, 100 nM) and incubated at 37 °C, 5% CO_2_ for 48 hr. The cells were then washed three times (centrifugation at 800 × g) with antibiotic-free growth medium and challenged with 200 µL C. trachomatis strain K (100 µg) for 60 mins. Afterwards, medium containing excess bacteria was replaced with antibiotic-free growth medium and infected cells were incubated at 37 °C, 5% CO_2_ for 24 hr. At the end of incubation, cells were washed with HBSS, fixed with 100% methanol (Thermo Fisher, Ontario, Canada) for 10 min and blocked with 3% BSA (Thermo Fisher, Ontario, Canada) for 60 min at room temperature. Staining was conducted with 200 µL FITC-anti-chlamydia lipopolysaccharide antibody (B410F) (1:5 dilution) (Thermo Fisher, Ontario, Canada) overnight at 4 °C. Images were taken using fluorescence microscopy (Nikon, ECLIPSE Ti). Intracellular C. trachomatis RB foci were quantified by Image J as described previously^58^.

The amount of extracellular C. trachomatis released into the cell culture supernatant was quantified by Primerdesign genesig Kit for C.trachomatis genomes (Genesig, Pennsylvania, USA) using qRT-PCR. Primers and probe sequences in this kit have 100% homology with a broad range of C. trachomatis sequences. VK2/E6E7 cells were infected the same way as described previously and the supernatant containing released C. trachomatis was collected 24 hr post infection. DNA was extracted using Genesig Easy DNA/RNA extraction kit (Genesig, Pennsylvania, USA) and quantification of extracted DNA was performed using Precision®PLUS 2X qPCR Master Mix (Genesig, Pennsylvania, USA). A primer and probe mix provided by the kit was used to detect an endogenous gene for normalization of the results. The thermal cycle conducted was as follows: a run of 50 cycles at 95 °C for 2 min for the enzyme activation, 95 °C for 10 s for the denaturation and 60 °C for 60 s for the data collection through the FAM channel.

### Statistical analysis

Data are presented as mean +/−standard deviation (SD). Unless specified, non-parametric One-way ANOVA (Tukey test) was used for multiple comparisons with p < 0.05 considered to be significant. GraphPad Prism 6 was the software used for conducting statistical analysis.

## Electronic supplementary material

Supplementary Information

## Data Availability

The datasets generated during and/or analysed during the current study are available from the corresponding author on reasonable request.
