# Supplementary material for: Autophagy induction and PDGFR-β knockdown by siRNA-encapsulated nanoparticles reduce chlamydia trachomatis infection
Source: Sci Rep. 2019 Feb 4;9:1306. doi: 10.1038/s41598-018-36601-y (PMC6361950; doi:10.1038/s41598-018-36601-y)
Supplement: Supplementary file 1 — Supplementary Information [file 41598_2018_36601_MOESM1_ESM.doc]

**Autophagy induction and PDGFR-b knockdown by siRNA-encapsulated nanoparticles reduce chlamydia trachomatis infection**

**Sidi Yang, Yannick Traore, Celine Jimenez, and Emmanuel A. Ho^*^**

**Table S1**. P values of the statistical analysis for autophagy-regulatory genes

Column A: Naïve control

Column B: Nonsilencing siRNA PLGA-PEG NP

Column C: Nonsilencing siRNA-PEI-PLGA-PEG NP

Column D: PDGFR-β siRNA-PEI-PLGA-PEG NP

| **Beclin-1** | | | | | |
| --- | --- | --- | --- | --- | --- |
| Tukey's multiple comparisons test | Mean Diff. | 95% CI of diff. | Significant? | Summary | Adjusted P Value |
|  |  |  |  |  |  |
| Column A vs. Column B | -0.02583 | -0.2868 to 0.2352 | No | ns | 0.9882 |
| Column A vs. Column C | -0.3769 | -0.6379 to -0.1159 | Yes | ** | 0.0074 |
| Column A vs. Column D | -0.8869 | -1.148 to -0.6259 | Yes | **** | < 0.0001 |
| Column B vs. Column C | -0.3511 | -0.6121 to -0.09010 | Yes | * | 0.0111 |
| Column B vs. Column D | -0.8611 | -1.122 to -0.6001 | Yes | **** | < 0.0001 |
| Column C vs. Column D | -0.51 | -0.7710 to -0.2490 | Yes | ** | 0.0011 |
| **VPS34** | | | | | |
| Tukey's multiple comparisons test | Mean Diff. | 95% CI of diff. | Significant? | Summary | Adjusted P Value |
|  |  |  |  |  |  |
| Column A vs. Column B | -0.465 | -1.056 to 0.1266 | Yes | * | 0.0498 |
| Column A vs. Column C | -1.195 | -1.787 to -0.6040 | Yes | *** | 0.0009 |
| Column A vs. Column D | -1.373 | -1.964 to -0.7811 | Yes | *** | 0.0003 |
| Column B vs. Column C | -0.7305 | -1.322 to -0.1390 | Yes | * | 0.0177 |
| Column B vs. Column D | -0.9077 | -1.499 to -0.3162 | Yes | ** | 0.0051 |
| Column C vs. Column D | -0.1772 | -0.7687 to 0.4144 | No | ns | 0.7753 |
| **TECPR-1** | | | | | |
| Tukey's multiple comparisons test | Mean Diff. | 95% CI of diff. | Significant? | Summary | Adjusted P Value |
|  |  |  |  |  |  |
| Column A vs. Column B | 0.2693 | -0.07488 to 0.6135 | No | ns | 0.1334 |
| Column A vs. Column C | -0.345 | -0.6892 to -0.0007872 | Yes | * | 0.0495 |
| Column A vs. Column D | -0.4237 | -0.7679 to -0.07945 | Yes | * | 0.018 |
| Column B vs. Column C | -0.6143 | -0.9585 to -0.2701 | Yes | ** | 0.002 |
| Column B vs. Column D | -0.693 | -1.037 to -0.3488 | Yes | *** | 0.0009 |
| Column C vs. Column D | -0.07867 | -0.4229 to 0.2655 | No | ns | 0.8817 |
| **UVRAG** | | | | | |
| Tukey's multiple comparisons test | Mean Diff. | 95% CI of diff. | Significant? | Summary | Adjusted P Value |
|  |  |  |  |  |  |
| Column A vs. Column B | 0.0502 | -0.2091 to 0.3095 | No | ns | 0.9229 |
| Column A vs. Column C | -0.3793 | -0.6387 to -0.1200 | Yes | ** | 0.0068 |
| Column A vs. Column D | -0.3392 | -0.5985 to -0.07984 | Yes | * | 0.013 |
| Column B vs. Column C | -0.4295 | -0.6889 to -0.1702 | Yes | ** | 0.0032 |
| Column B vs. Column D | -0.3894 | -0.6487 to -0.1300 | Yes | ** | 0.0059 |
| Column C vs. Column D | 0.04016 | -0.2192 to 0.2995 | No | ns | 0.9578 |

| No. | Total LC3B | Initiation stage of autophagy (formation of autophagosome, markers: Beclin-1 and VPS 34) | Degradation stage of autophagy (degradation of autophagosome by forming autolysosome, the compartment for degradation markers: TECPR-1 and UVRAG) | Autophagic flux |
| --- | --- | --- | --- | --- |
| 1 | ↑ | ↑ | − | − |
| 2 | ↑ | − | ↓ | ↓ |
| 3 | ↑ | ↑ | ↓ | ↓ |
| 4 | ↑ | ↑↑ | ↑ | ↑ |
| 5 | ↑ | ↓ | ↓↓ | ↓ |
| 6 | − | ↑↑ | ↑↑ | ↑↑ |
| 7 | − | − | − | − |
| 8 | ↓ | ↓ | − | ↓ |
| 9 | ↓ | − | ↑ | ↑ |
| 10 | ↓ | ↓ | ↑ | ↑ or ↓ |
| 11 | ↓ | ↑ | ↑↑ | ↑ |
| 12 | ↓ | ↓↓ | ↓ | ↓ |

# Table S2. Changes in total LC3B with all possible changes in initiation stage & degradation stage of autophagy and autophagic flux.

**Table S3**. Sequences of primers and siRNAs

| PDGFR-β primer | forward: 5’-ACCATTCCATGCCGAGTAACA-3’  reverse: 5’-CTGTCCCCAATGGTGGTTTT-3’ |
| --- | --- |
| GAPDH primer | forward: 5’-AAGAAGGTGGTGAAGCAGGCG-3’  reverse: 5’-AGACAACCTGGTCCTCAGTGTAGC-3’ |
| Beclin-1 primer | forward: 5’-ACCGCAAGATAGTGGCAGAAA-3’  reverse: 5’-GGGCATAACGCATCTGGTTT-3’ |
| UVRAG primer | forward: 5’-GGCAAACCCTTCCCAACCT-3’  reverse: 5’-TCTGCACCCCCAAATATGGA-3’ |
| TECPR-1 prmier | forward: 5’-CCCGCCACCTACACGAAA-3’  reverse: 5’-CCCCTACAGAGAGGTCGTTGAA-3’ |
| VPS-34 primer | forward: 5’-GGAAAAGCAGTGCCTGTAGGA-3’  reverse: 5’-GGCAAGACGGCTCATCTGAT-3’ |
| PDGFR-β siRNA | sense 5'-GAAAGGAGACGUCAAAUAUdTdT-3'  antisense 3'-dTdTCUUUCCUCUGCAGUUUAUA-5' |
